# Trimester-Specific Gestational Weight Gain and Infant Size for Gestational Age

**DOI:** 10.1371/journal.pone.0159500

**Published:** 2016-07-21

**Authors:** Sneha B. Sridhar, Fei Xu, Monique M. Hedderson

**Affiliations:** Division of Research, Kaiser Permanente Northern California, Oakland, California, United States of America; University of Tennessee Health Science Center, UNITED STATES

## Abstract

Gestational weight gain is known to influence fetal growth. However, it is unclear whether the associations between gestational weight gain and fetal growth vary by trimester. In a diverse cohort of 8,977 women who delivered a singleton between 2011 and 2013, we evaluated the associations between trimester-specific gestational weight gain and infant size for gestational age. Gestational weight gain was categorized per the 2009 Institute of Medicine (IOM) recommendations; meeting the recommendations was the referent. Large for gestational age and small for gestational age were defined as birthweight > 90^th^ percentile or <10^th^ percentile, respectively, based on a national reference standard birthweight distribution. Logistic regression models estimated the odds of having a large or small for gestational age versus an appropriate for gestational age infant. Only gestational weight gain exceeding the IOM recommendations in the 2^nd^ and 3^rd^ trimesters independently increased the odds of delivering a large for gestational age infant (Odds Ratio (95% Confidence Interval): 1^st^: 1.17 [0.94, 1.44], 2^nd^: 1.47 [1.13, 1.92], 3^rd^: 1.70 [1.30, 2.22]). Gestational weight gain below the IOM recommendations increased the likelihood of having a small for gestational age infant in the 2^nd^ trimester only (1.76 [1.23, 2.52]). There was effect modification, and gestational weight gain below the IOM recommendations increased the likelihood of having a small for gestational age infant in the 2^nd^ trimester and only among women with a pre-pregnancy body mass index from 18.5–24.9 kg/m^2^ (2.06 [1.35, 3.15]). These findings indicate that gestational weight gain during the 2^nd^ and 3^rd^ trimesters is more strongly associated with infant growth. Interventions to achieve appropriate gestational weight gain may optimize infant size at birth.

## Introduction

Gestational weight gain (GWG) is a known driver of fetal growth. In 2009, the Institute of Medicine (IOM) released updated guidelines on GWG in part to account for the increasing prevalence of obesity among women of reproductive age. The IOM report highlighted the need for further research on how patterns of GWG throughout the course of pregnancy impact neonatal outcomes. Infants born either LGA or SGA may be more likely to accumulate excess fat in early childhood [1] and are more likely to become obese later in life [2–4]. However, it is currently unclear whether there are critical windows in pregnancy when GWG most strongly influences fetal growth.

Only two prior studies looked at GWG in all three trimesters and birthweight in the context of the current obesity epidemic, and none specifically examined size for gestational age, which is an important predictor of later life obesity [5,6]; therefore, we need to clarify the associations of patterns of GWG throughout the entire course of pregnancy on infant size at birth. Additionally, most prior studies lacked information on measured pre-pregnancy weight and were unable to determine 1^st^ trimester GWG. A better understanding of the timing of when GWG influences growth will help inform future lifestyle interventions designed to optimize fetal growth. The aim of this cohort study was to determine whether trimester-specific gestational weight gain impacts infant size for gestational age and to explore whether maternal pre-pregnancy body mass index (BMI) modifies this relationship.

## Materials and Methods

The study setting was Kaiser Permanente Northern California, a large group practice prepaid health plan which provides comprehensive medical services to members residing in a 14-county region of Northern California (approximately 30% of the surrounding population). The demographic, racial/ethnic, and socioeconomic makeup of the Kaiser Permanente Northern California membership is well representative of the population residing in the same geographic area, except that the very poor and the very wealthy are under-represented [7,8].

### Cohort identification

We identified 12,662 female Kaiser Permanente Northern California members who completed a detailed health survey as part of the Kaiser Permanente Research Program on Genes, Environment, and Health (RPGEH) between 2007 and 2012 and delivered a singleton livebirth between 2011 and 2013. Pregnancies were identified through the electronic medical record (EMR). We then excluded 163 women with preexisting diabetes (either type 1 or type 2) and limited the cohort to full-term births (gestational age ≥ 37 weeks), resulting in 11,643 women. We restricted the cohort to women who had a pre-pregnancy weight as well as a final pregnancy weight within four weeks of delivery available (n = 10,285). We then limited the cohort to women whose deliveries occurred after the RPGEH health survey was completed and for whom infant birthweight information was available (n = 9,153). Due to the small number of underweight women (BMI < 18.5 kg/m^2^) (n = 176), they were excluded from the cohort. The final analytic cohort consisted of 8,977 women.

This study was approved by the Kaiser Permanente Northern California Institutional Review Board, who waived the requirement for obtaining written informed consent from study participants. Patient records were anonymized and de-identified prior to analysis.

### Maternal characteristics

Self-reported maternal race/ethnicity [Non-Hispanic white, hereafter referred to as white, 2) African American, 3) Asian/Pacific Islander, 4) Hispanic, and 5) Other/Unknown] and educational attainment were obtained from the RPGEH survey.

### Pre-pregnancy dietary pattern

The RPGEH survey included 20 food categories, including sugar-sweetened beverages. To identify major dietary patterns, principal components analysis was used on the 20 foods to identify factors that accounted for much of the variance. The food groups (factors) were rotated using an orthogonal transformation, resulting in uncorrelated, independent factors. The factor score for each factor (pattern) was calculated by summing intakes of food groups weighted by factor loading, and each individual was assigned a score for each identified pattern. Individuals with a high score for a pattern compared with individuals with lower scores have a stronger tendency to follow that pattern. We identified two distinct dietary patterns: Prudent and Western. The dietary pattern scores were then categorized by tertiles.

### Pre-pregnancy physical activity

Volume of total METs was calculated as minutes per week based on four questions. The first three questions assessed the following forms of physical activity during the previous 7 days: walking, moderate recreational activity and vigorous recreational activity. A fourth question assessed sedentary behaviors. Women reported the average minutes per week she spent doing each activity. These questions were adopted from validated scales.

### Gestational diabetes

Gestational diabetes status was assessed through the Kaiser Permanente Northern California Pregnancy Glucose Tolerance Registry [9] and defined as having at least two plasma glucose values on the 100-g, 3-hour oral glucose tolerance test meeting or exceeding the Carpenter-Coustan thresholds [10].

### Exposure ascertainment

We searched the EMR data for a pre-pregnancy weight measured within 12 months of conception. For those missing a measured pre-pregnancy weight (19.0%), a self-reported pre-pregnancy weight was used for 15.0%, and an early pregnancy measured weight was used for 4.0%. To validate this method of estimating pre-pregnancy weight, we compared the self-reported pre-pregnancy weight to a weight measured within 12 months of the last menstrual period among the 4,723 women for whom both measurements were available. The intra-class correlation coefficient between the two weights was 0.975.

Pre-pregnancy BMI was calculated as pre-pregnancy weight (kilograms) divided by height (meters) squared. BMI categories were created in accordance with the 2009 Institute of Medicine (IOM) GWG recommendations as follows: normal weight (18.5–24.9 kg/m^2^), overweight (25.0–29.9 kg/m^2^), and obese (≥30.0 kg/m^2^). Total gestational weight gain was calculated as the difference between the last measured pregnancy weight and pre-pregnancy weight, in kilograms; the last weight measured during pregnancy was obtained from the EMR and measured within four weeks of delivery.

We calculated 1^st^ trimester GWG to be the difference between the last available weight measurement during the 1^st^ trimester (gestational weeks, mean (SD): 13.6 (1.3)) and pre-pregnancy weight. Among the 8,977 women in the cohort, 78.5% women had a 1^st^ trimester weight measurement. We calculated 2^nd^ trimester GWG rate as the difference between the early 3^rd^ trimester weight (gestational weeks, mean (SD): 29.9 (1.3)) and the last 1^st^ trimester weight measurement, divided by the gestational weeks between the two measurements. Finally, we calculated 3^rd^ trimester GWG rate to be the difference between the last pregnancy weight (gestational weeks, mean (SD): 38.7 (1.3)) and the early 3^rd^ trimester weight measurement, divided by the gestational weeks between the two measurements.

The 2009 IOM recommendations suggest a range of absolute weight gain in the first trimester and a range of pregravid BMI-specific rates of weight gain per week for the 2^nd^ and 3^rd^ trimesters [11]. We determined whether each woman met, exceeded or was below the IOM recommendations for trimester-specific weight gain, by calculating whether she met recommended absolute amount of weight gain in the first trimester (1.1–4.4 pounds) for all women. For the 2^nd^ and 3^rd^ trimester, we determined whether the rate of GWG in each respective trimester met the IOM range of recommended weight gain for her pre-pregnancy BMI.

For total GWG weight gain, we determined whether each woman met, exceeded or was below the IOM recommendations based on the last measured weight in pregnancy minus her pregnancy weight. We subtracted 13 weeks from the gestational age at the delivery and multiplied this value by the BMI-specific recommended rate of weight gain for the 2nd and 3rd trimesters. Weight gained after the first trimester was then added to the BMI-specific absolute weight gain recommended by the IOM for the first trimester.

### Offspring characteristics

We used sex- and gestational age-specific references for size for gestational age from Oken et al. [12], a United States national reference standard. Size for gestational age was categorized as follows: large for gestational age if birthweight greater than the 90^th^ percentile, small for gestational age if birthweight less than the 10^th^ percentile, and appropriate for gestational age if birthweight between the 10^th^ and 90^th^ percentiles, inclusive. Sex and birth weight data for the children were obtained from the EMR.

### Statistical analysis

Unconditional logistic regression analysis was used to obtain odds ratios (ORs) and confidence intervals (CIs) estimating the odds of a small or large for gestational age infant (both versus appropriate for gestational age) associated with maternal GWG for both total GWG and trimester-specific GWG, as well as a single model including trimester-specific GWG in all three trimesters simultaneously. We estimated the odds of LGA or SGA associated with exceeding and gaining below the recommendations, as compared to meeting the recommendations.

Covariates in the fully adjusted model included those of a priori interest based on existing literature (maternal age at delivery, race/ethnicity, gestational age at delivery, parity (number of previous livebirths), pre-pregnancy BMI, gestational diabetes status, infant sex (with male as the referent), pre-pregnancy physical activity (in tertiles of total MET-minutes/week, with the lowest tertile as the referent), pre-pregnancy Western dietary pattern score (in tertiles, with the lowest tertile as the referent), and pre-pregnancy Prudent dietary pattern score (in tertiles, with the lowest tertile as the referent).

To assess the potential modifying effect of maternal pre-pregnancy BMI in each trimester, we included separate cross product (interaction) terms in each regression model, using trimester-specific gestational weight gain (Exceeded vs Met/Below) as the exposure. Finally, we conducted the following sensitivity analyses: 1) excluding women with self-reported weights, and 2) excluding women with conditions that may affect gestational weight gain. SAS version 9.3 (SAS Institute Inc., Cary, NC) was used for all analyses.

## Results

Table 1 summarizes the demographic characteristics of the 8,977 women in the cohort. Women were, on average, 33 years old at delivery, and the majority (65.6%) had the equivalent of a college degree (Table 1). The cohort was diverse (43.0% were racial/ethnic minorities), and 63.9% were multiparous, while 46.6% were overweight or obese. The proportions of SGA, AGA and LGA infants were 8.4%, 81.6%, and 10.0%, respectively. Overall, 11.1% fell below, 22.8% met and 66.1% exceeded the IOM recommendations for total GWG; however, the percent exceeding the IOM recommendations for rate of GWG in each trimester were quite a bit higher (1^st^: 46.9%, 2^nd^: 72.3%, 3^rd^: 63.6%) (Table 2).

**Table 1:** Characteristics of the 8,977 Pregnant Women at Kaiser Permanente Northern California Who Delivered Between 2011 and 2013.

Characteristic	No.	%
***Maternal***		
** Education (years)**		
High School Graduate or Less	1,079	12.0
Some College	1,570	17.5
College Graduate or Higher	5,885	65.6
Other/Unknown	443	4.9
** Race/Ethnicity**		
Non-Hispanic White	4,626	51.5
African American	340	3.8
Asian/Pacific Islander	1,927	21.5
Hispanic	1,585	17.7
Other/Unknown	499	5.6
** Parity**		
0	3,204	35.7
1	3,712	41.4
≥ 2	2,015	22.5
Unknown	46	0.5
**Pre-pregnancy body mass index (kg/m**^**2**^**)**		
18.5–24.9 (Normal Weight)	4,798	53.5
25.0–29.9 (Overweight)	2,435	27.1
≥ 30.0 (Obese)	1,744	19.4
** Had gestational diabetes**	624	7.0
***Child***		
** Male infant sex**	4, 566	50.9
** Size for gestational age**		
Small for gestational age	756	8.4
Appropriate for gestational age	7,322	81.6
Large for gestational age	899	10.0
	Mean (SD)	
** Maternal age at delivery (years)**	33.0 (4.8)	
** Infant birth weight (grams)**	3,495.6 (460.3)	
** Infant’s gestational age at delivery (weeks)**	39.2 (1.1)	
** Pre-pregnancy volume of physical activity, MET-mins/week**	799.2 (912.0)	

**Table 2:** Adjusted^a^ Odds Ratios (ORs) and 95% Confidence Intervals (CIs) for Size for Gestational Age Associated With Total and Trimester-Specific Gestational Weight Gain (GWG).

*Pregnancy Risk Factor*	N (%)	Small for gestational age (SGA)		Large for gestational age (LGA)	
**Total**		OR	95% CI	OR	95% CI
IOM GWG Recommendations					
Below	995 (11.08)	**1.55**	**[1.17, 2.07]**	**0.58**	**[0.41, 0.81]**
Met	2,049 (22.82)	Reference		Reference	
Exceeded	5, 933 (66.09)	**0.64**	**[0.51, 0.80]**	**2.12**	**[1.75, 2.58]**
**1**^**st**^ **Trimester**					
IOM GWG Recommendations					
Below	2,311 (32.79)	1.02	[0.76, 1.36]	0.80	[0.63, 1.01]
Met	1,432 (20.32)	Reference		Reference	
Exceeded	3,305 (46.89)	0.82	[0.62, 1.08]	1.17	[0.94, 1.44]
**2**^**nd**^ **Trimester**					
IOM GWG Recommendations					
Below	781 (12.92)	**1.76**	**[1.23, 2.52]**	0.79	[0.54, 1.15]
Met	893 (14.78)	Reference		Reference	
Exceeded	4,369 (72.30)	**0.70**	**[0.51, 0.95]**	**1.47**	**[1.13, 1.92]**
**3**^**rd**^ **Trimester**					
IOM GWG Recommendations					
Below	1,719 (23.90)	0.98	[0.71, 1.36]	0.93	[0.68, 1.27]
Met	900 (12.52)	Reference		Reference	
Exceeded	4,572 (63.58)	0.81	[0.60, 1.09]	**1.70**	**[1.30, 2.22]**

^a^Adjusted for race/ethnicity, maternal age at delivery, gestational age at delivery, pre-pregnancy BMI (kg/m^2^), gestational diabetes status, infant sex, parity, pre-pregnancy physical activity (in tertiles of MET-mins/week), pre-pregnancy Prudent dietary pattern score (in tertiles), and pre-pregnancy Western dietary pattern score (in tertiles).

### Total GWG

Table 2 displays the odds ratios (ORs) and 95% confidence intervals (CIs) associated with SGA and LGA infants, by trimester-specific rate of GWG and total rate of GWG.

Overall, women who gained below the IOM recommendations for total GWG were more likely to deliver an SGA infant (OR (95% CI): 1.55 [1.17, 2.07]) and less likely to deliver an LGA infant (0.58 [0.41, 0.81]), compared to women with met the IOM recommendations. In contrast, women who exceeded the IOM recommendations were less likely to deliver SGA infants and more likely to deliver LGA infants (SGA: 0.64 [0.51, 0.80]; LGA: 2.12 [1.75, 2.58]).

### Trimester-specific GWG

In models assessing each trimester separately (Table 2), excess GWG increased the likelihood of an LGA infant in the 2^nd^ and 3^rd^ trimesters only (1^st^: 1.17 [0.94, 1.44], 2^nd^: 1.47 [1.13, 1.92], 3^rd^: 1.70 [1.30, 2.22]). In the second trimester only, excess GWG was associated with decreased risk of having an SGA infant, and gaining below the IOM recommendations was associated with increased risk of an SGA infant.

**Fig 1:**
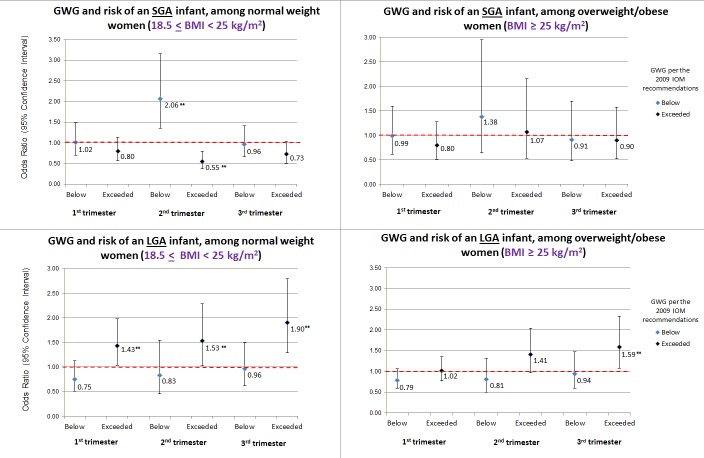
Odds ratios and 95% confidence intervals from multivariable* models of trimester-specific GWG and risk of SGA or LGA infants, by maternal pre-pregnancy BMI. *Adjusted for race/ethnicity, maternal age at delivery, gestational age at delivery, parity, pre-pregnancy BMI, gestational diabetes status, infant sex, pre-pregnancy physical activity (in tertiles of MET-minutes/week), pre-pregnancy Western dietary pattern score (in tertiles), and pre-pregnancy Prudent dietary pattern score (in tertiles); ** = indicates statistical significance (p-value <0.05).

There was significant effect modification by maternal pre-pregnancy BMI for 2^nd^ trimester GWG and SGA only (P-value interaction term = 0.0016); there was no significant effect modification by BMI for SGA in the 1^st^ or 3^rd^ trimester, as well as during any trimester for GWG and LGA. Normal weight women whose 2^nd^ trimester GWG was below the IOM recommendations had a twofold increased likelihood of having an SGA infant (2.06 [1.35, 3.15], while those with excess 2^nd^ trimester GWG were less likely to have an SGA infant (0.55 [0.37, 0.80]). In contrast, for overweight and obese women, GWG outside of the IOM recommendations was not associated with SGA (Fig 1). Excess GWG was associated with LGA in all three trimesters for normal weight women, and in the 3^rd^ trimester only for overweight and obese women.

In a model adjusting for all three trimesters simultaneously, excess GWG in the 2^nd^ and 3^rd^ trimesters was associated with having an LGA infant (1^st^: 1.09 (0.87–1.37, 2^nd^: 1.33 (1.01–1.74), 3^rd^: 1.63 (1.21–2.20)). There was no significant association during any trimester between GWG below the IOM recommendations and having an LGA infant (1^st^: 0.78 (0.61–1.00), 2^nd^: 0.81 (0.55–1.17), 3^rd^: 1.04 (0.74–1.46)). In the 2^nd^ trimester only, GWG below the IOM recommendations was associated with increased odds of an SGA infant (1^st^: 1.07 (0.78–1.46), 2^nd^: 1.74 (1.22–2.49), 3^rd^: 1.00 (0.70–1.44)), while excess 2^nd^ GWG was associated with decreased odds of an SGA infant (1^st^: 0.88 (0.66–1.18), 2^nd^: 0.71 (0.52–0.99), 3^rd^: 0.91 (0.65–1.27)).

Results were also similar for the sensitivity analysis that was restricted to women with a measured weight (n = 7,285) as well as for a separate sensitivity analysis that excluded 1,781 women with certain conditions that may impact gestational weight gain (gestational diabetes, bariatric surgery, any thyroid disorder, and preeclampsia or gestational hypertension) (data not shown).

## Discussion

Overall, gaining below the current IOM GWG recommendations increased the risk of SGA and decreased the risk of LGA. In contrast, excess GWG increased the risk of LGA and decreased the risk of SGA. These associations varied by trimester. Only excess rate of GWG in the 2^nd^ and 3^rd^ trimesters significantly increased risk of LGA, and GWG below the IOM recommendation significantly increased risk of SGA in the 2^nd^ trimester only. We found significant effect modification by pre-pregnancy BMI; the association between 2^nd^ trimester GWG below the IOM recommendations and SGA was only significant among normal weight women. Excess GWG was associated with LGA in all three trimesters among normal weight women, but only in the 3^rd^ trimester among overweight/obese women.

There is some evidence from previous studies that in comparison to the 1^st^ trimester, the 2^nd^ and/or 3^rd^ trimesters may form the period when GWG most strongly influences fetal growth and birth weight [13–18]. Gaillard et al found a positive association between standard deviation of change in GWG per week and size for gestational age in all three trimesters, but the strongest associations were in the 2^nd^ and 3^rd^ trimesters [19]. In a prospective cohort study, Margerison-Zilco et al found that that both total GWG and GWG in all three trimesters were positively associated with birthweight, with the impact stronger, though not significantly, in the 2^nd^ trimester [14]. Abrams et al found that maternal GWG was positively associated with birthweight in all three trimesters, with the strongest association occurring in the 2^nd^ trimester [13].

We found that in the 2^nd^ trimester only, gaining below the IOM recommendations was associated with having an SGA infant among normal weight women (pre-pregnancy BMI: 18.5–24.9 kg/m^2^), but not among overweight and obese women. Consistent with our findings, Drehmer et al found an overall association between GWG below the IOM recommendations and SGA in the 2^nd^, but not the 3^rd^ trimester, but they did not look at the association stratified by pre-pregnancy BMI [20]. Additionally, while excess GWG was associated with LGA in all three trimesters for normal weight women, only excess 3^rd^ trimester GWG was associated with LGA for overweight and obese women. A prior study found that GWG in the 2nd and 3rd trimesters of pregnancy, but not first trimester, was associated with risk of large for gestational age infants; however, they did not examine the associations by pre-pregnancy BMI [21]. Previous research [22] has found that the association between maternal GWG and birthweight is attenuated in obese women compared with normal weight women, suggesting that GWG is not as important of a contributor to fetal growth in obese versus non-obese women.

One possible consequence of excess GWG is the transfer of excess nutrients across the placenta; the influx of excess nutrients, particularly maternal glucose and lipids/fatty acids, can accelerate fetal growth and fat accretion and lead to an LGA infant [23,24]. On the other hand, calorie restriction and maternal undernutrition are thought to impede fetal growth; thus, it is biologically plausible for excess GWG to reduce the risk of having an SGA infant, and for low GWG to increase the risk of SGA. We found that the impact of GWG on infant size for gestational age varied by trimester, which may be due in part to the fact that both the composition of GWG and the biological development of the fetus differ by trimester [25,26]. GWG during the first trimester is disproportionately maternal fat and may influence maternal glucose metabolism later in pregnancy [25,27]. The fetal-placental glucose utilization rates peak in the 2^nd^ trimester [28], and excess GWG may further accelerate glucose utilization and thereby increase fetal growth. The 3^rd^ trimester is when the placenta most actively shunts nutrients to the fetus, and the majority of fetal growth [29] and fat accretion [30] occurs; therefore, it is unsurprising that 3^rd^ trimester GWG was associated with fetal growth and infant size at birth.

The strengths of the present study include the large, diverse cohort, the historical prospective cohort study design, and the availability of measured pre-pregnancy weight in the majority of participants (81%), as well as our ability to assess GWG in all three trimesters. We were also able to adjust for pre-pregnancy dietary pattern and physical activity; although both measures were assessed before pregnancy, they are likely a good surrogate for women’s lifestyle during pregnancy. However, one limitation is that the diet and physical activity components of the RPGEH survey are not validated measures. We also lacked information on the composition of maternal weight gain (e.g., fat, placental weight, fluid, etc.), as well as information on infant body composition at birth, which may be important given that neonatal adiposity may be a stronger predictor of subsequent obesity factors than size for gestational age alone [31,32].

In summary, GWG according to the 2009 IOM recommendations is associated with size for gestational age, and our data suggest that the timing of the GWG may be important, as associations between excess GWG and LGA appear to be stronger in the 2^nd^ and 3^rd^ trimesters. We also found that there was no significant association in any trimester between GWG below the current recommendations and SGA among women who are overweight or obese before pregnancy. This suggests that perhaps less weight gain may be safe for overweight and obese women, but this needs to be confirmed in future studies that include other outcomes as well. Clarifying how and when GWG is driving fetal growth will help to inform future prevention strategies.
